# The Effect of Conformational Variability of Phosphotriesterase upon *N*-acyl-L-homoserine Lactone and Paraoxon Binding: Insights from Molecular Dynamics Studies

**DOI:** 10.3390/molecules181215501

**Published:** 2013-12-12

**Authors:** Dongling Zhan, Zhenhuan Zhou, Shanshan Guan, Weiwei Han

**Affiliations:** 1 Key Laboratory for Molecular Enzymology and Engineering of the Ministry of Education, Jilin University, Changchun 130023, China; E-Mail: zdlgale@126.com; 2 College of Food Science and Engineering, Jilin Agricultural University, Changchun 130118, China; 3 Jilin Provincial Research Institute of Population and Life sciences, Changchun 130041, China; E-Mail: hanbx4@126.com; 4 State Key Laboratory of Theoretical and Computational Chemistry, Jilin University, Changchun 130023, China; E-Mail: guanss12@mails.jlu.edu.cn

**Keywords:** PTE, homology modeling, molecular docking, molecular dynamics

## Abstract

The organophosphorous hydrolase (PTE) from *Brevundimonas diminuta* is capable of degrading extremely toxic organophosphorous compounds with a high catalytic turnover and broad substrate specificity. Although the natural substrate for PTE is unknown, its loop remodeling (loop 7-2/H254R) led to the emergence of a homoserine lactonase (HSL) activity that is undetectable in PTE (k_cat_/k_m_ values of up to 2 × 10^4^), with only a minor decrease in PTE paraoxonase activity. In this study, homology modeling and molecular dynamics simulations have been undertaken seeking to explain the reason for the substrate specificity for the wild-type and the loop 7-2/H254R variant. The cavity volume estimated results showed that the active pocket of the variant was almost two fold larger than that of the wild-type (WT) enzyme. pKa calculations for the enzyme (the WT and the variant) showed a significant pKa shift from WT standard values (ΔpKa = 3.5 units) for the His254residue (in the Arg254 variant). Molecular dynamics simulations indicated that the displacement of loops 6 and 7 over the active site in loop 7-2/H254R variant is useful for N-acyl-L-homoserine lactone (C4-HSL) with a large aliphatic chain to site in the channels easily. Thence the expanding of the active pocket is beneficial to C4-HSL binding and has a little effect on paraoxon binding. Our results provide a new theoretical contribution of loop remodeling to the rapid divergence of new enzyme functions.

## 1. Introduction

Organophosphorous compounds (OP) are efficient insecticides utilized for more than 70 years for protecting agricultural crops [1,2,3,4,5,6]. However, the extensive use of organophosphorus pesticides can be cause a lot of direct or indirect toxic effects. Because they are a serious threat to the environment and human health, it is important to develop of a cost-effective way to remove them from the environment. Currently organophosphate poisoning treatment involves the use of chemicals (atropine and pralidoxime), but their side effects (excessive use can cause poisoning again) are very difficult to overcome [7,8]. 

OP-degrading enzymes, with their broad activity against diverse OP pesticides *in vitro* and *in vivo* [9,10,11,12,13,14], and against several G-type nerve agents (sarin, soman, tabun) [15] have attracted researcher’s attention. The enzyme organophosphorus hydrolase (PTE; EC 3.1.8.1) from the Gram-negative bacterium *Brevundimonas diminuta* (PDB Id 1HZY) hydrolyzes OP compounds with high catalytic efficiency [16]. In addition, PTE has also been shown to catalyze the detoxification of chemical warfare agents such as sarin (GB), soman (GD), and VX [16,17]. From X-ray crystallographic analyses, each subunit of PTE is known to adopt a “TIM” barrel motif with the binuclear metal center located at the C-terminal portion of the *β*-barrel [16,18,19,20]. In the wild-type enzyme, the two zinc ions are ligated to the protein through the side chains of a single aspartate (Asp301) and four histidines (His55, His57, His201, and His230) [16]. In addition, the two metal ions are bridged together by a carbamate functional group, formed by the carboxylation of Lys169, and a water (or hydroxide ion) from the solvent. The active site of PTE has previously been denoted as the small, large, and leaving group pockets and are defined by the space enclosed by the side chains of Gly60, Ile106, Leu303, and Ser308; side chains of His254, His257, Leu271, and Met317; and side chains of Trp131, Phe132, Phe306, and Tyr309, respectively [16,21,22,23].

**Figure 1:**
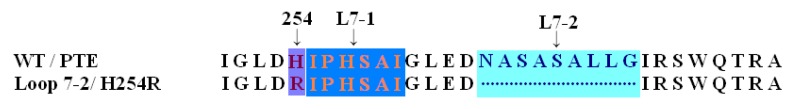
Sequence alignment between the WT PTE and loop7-2/H254R.

It was reported that PTE exhibits a weak, promiscuous lactonase activity that could be a vestige of its PTE-like lactonase (PLL) ancestor, although PTE only share ~30% sequence identity with PLLs [24]. Tawfik *et al*. found that PTE has two closely related clades: PLL-A, which hydrolyzes N-acyl-L-homoserine lactone (HSLs) with different N-acyl chains and PLL-B which hydrolyzes other γ- or even δ-lactones [24]. Most notably, PTE’s loop 7 is 14 amino acids longer than in PLL-As [25], and 11 amino acids longer than PLL-Bs. However, deletion of this 14-amino-acid stretch resulted in a soluble but completely inactive protein. Parallel attempts to transplant PTE’s loop 7 along with loop 8, or all eight active-site loops, into various PLLs resulted in an insoluble, or inactive, proteins [26,27]. Further examination of the sequence and structure alignments indicated that the 14-aminoacid insertion in PTE’s loop 7 actually consists of two individual insertions events separated by a 4-amino-acid spacer (annotated as L7-1 (residue 255-260) and L7-2 (residue 265-273) (see Figure 1). The two loop 7 segments differ in their structural features. Compared to L7-1, the L7-2 segment is solvent exposed, loosely packed, and exhibits fewer interactions with the rest of PTE’s structure [24]. These two parts of loop 7 could diverge independently from one another. Tawfik *et al.* remodeled active-site loops of PTE by exchange with PLL’s loop(s) resulted in nonfunctional proteins, and found that a deletion in L7-2 of PTE, combined with a mutation (H254R) can lead to the emergence of an HSLase activity that was undetectable in PTE (k_cat_/K_M_ values of up to 2 × 10^4^) [24]. At the meantime, a minor decrease in PTE’s paraoxonase activity appeared in the variant. But the structural difference between the wild type (WT) enzyme and the variant enzyme is still not known.

In this study, the variant type was built by homology modeling. Docking study and molecular dynamic method were used to explore the conformational variability of the WT type upon and the variant upon *N*-acyl-L-homoserine lactone (C4-HSL) and paraoxon binding. Further experimental and theoretical studies are still needed.

## 2. Results and Discussion

2.1 Protein Preparation

**Figure 2:**
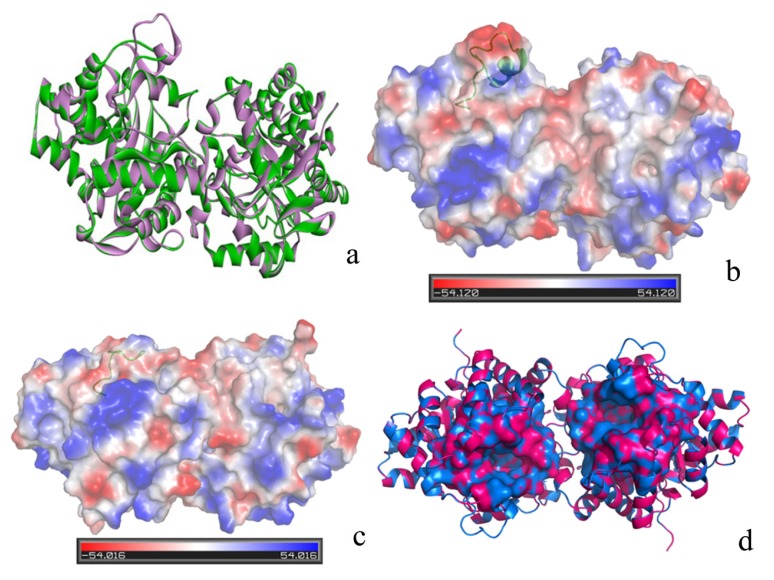
(**a**) The 3D structure alignment between the loop 7-2-H254R (green) and the WT PTE (purple); (**b**) The protein contact potential of the WT PTE generated with Pymol; (**c**) The protein contact potential of the loop 7-2-H254R variant; (**d**) The binding pocket between between the loop 7/H254R (purple) and the WT PTE (blue).

The 3D structure of the variant enzyme, loop 7-2-H254R, is shown in Figure 2a. The variant enzyme, loop 7-2-H254R, was similar to the WT enzyme, except for the loop 7 domain (the RMSD between the variant and the WT enzyme is 0.9 Å). The homology model was used for validation with Procheck [28], Verify_3D [29,30] and Errat [31]. The results are listed in Table 1. Seen from Table 1, the model was of high quality, with an above average QMEAN Z-score for a protein of its size (0.74), while the WT enzyme is 0.83. The second validation was carried out using Ramachandran plot calculations computed with the Procheck program by checking the detailed residue-by-residue stereo-chemical quality of a protein structure [28]. Altogether, 86.7% of all residues were in favored regions, and 100% of all residues were in allowed regions. In comparison with the homology model, the template, 7, WT PTE, had a similar Ramachandran plot 100% in the allowed regions. ERRAT is a so-called “overall quality factor” for nonbonded atomic interactions, and higher scores mean higher quality [31]. The normally accepted range is >50 for a high quality model. In the current case, the ERRAT score for the loop 7-2-H254R model is 98.722, well within the range of a high quality model, in the mean time the ERRAT score for the template is 99.379. Thus, the above analysis suggests that the backbone conformation and non-bonded interactions of homology model is all reasonable within a normal range. The final evaluation of the built loop 7-2/H254R structure was checked by Verify_3D [29,30]. It is to be noted that compatibility scores above zero correspond to acceptable side chain environment. 

We can see in the Table 1 that almost all residues in loop 7-2-/H254R are reasonable. All evaluations suggest that a reasonable homology model has been obtained that can be exposed for examination of protein-substrate interactions.

**Table 1:** Homology model validation with **QMEAN**, Procheck, Verify_3D and Errat.

Protein	QMEAN Z-Score	Procheck	Verify_3D	Errat
Loop7 -2/H254R	0.74	86.7% core 12.6% allow 0.7% gener 0.0% disall	96.90% of the residues had an averaged score > 0.2	98.722
PTE(PDB ID 1HZY)	0.83	89.6% core 9.7% allow 0.7% gener 0.0% disall	98.49% of the residues had an averaged score > 0.2	99.379

Figure 2b and 2c show the protein contact potential between the loop 7-2-H254R variant and the WT enzyme, respectively. There was no significant change of the protein contact potential between the loop 7-2-H254R variant and the WT enzyme. The cavity volume estimated by CASTp [32] is dependent on the radius of the probe sphere; a probe radius of 1.4 Å outlines a cavity of 1239 Å^3^ for loop 7-2-H254R variant , while a probe radius of 1.4 Å outlines a cavity of 651.5 Å^3^ for the WT PTE (seen Figure 2d). The different binding pocket site may influence the substrate specificity between the variant and the WT PTE.

**Figure 3:**
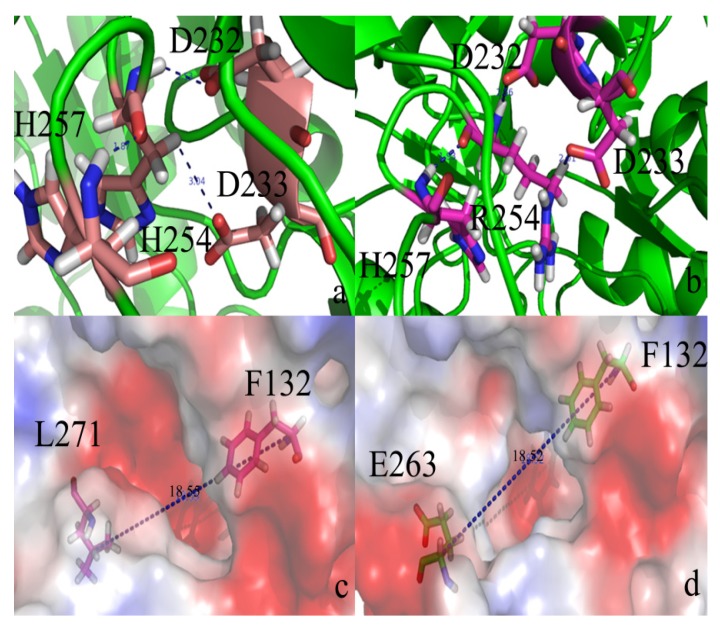
(**a**) H254 makes three hydrogen bonds with D232, D233, and H257, respectively in WT PTE; (**b**) R254 makes three hydrogen bonds with D232, D233, and H257, respectively in loop 7-2-H254R variant; (**c**) Leu271 and F132 function as the entrance gate in WT PTE; (**d**) Glu263 and F132 function as the entrance gate in loop 7-2-H254R variant.

Hydrogen bonds may be important in the substrate binding and catalytic efficiency. There are eight hydrogen bonds between residue 265 and residue 273 (listed in Table 2). In the loop 7/H254R variant, these hydrogen bonds are eliminated. H254 forms three hydrogen bond with Asp232, Asp233, and His257, respectively (seen Figure 3a). In the loop 7-2-H254R variant, the three hydrogen bonds are still present (seen Figure 3b). Leu271 is an important residue in the active pocket. Firstly, the large binding pocket contains Leu271. Secondly, Leu271 and Phe132 function as the entrance gate which allows for the controlled and selective passage of different substrates across the channel (Figure 3c). However, in the PTE variant, Glu263 and F132 became the entrance gate (Figure 3d). The distance between the Cα of Leu271 and the Cα of Phe132 was 18.55 Å in the WT PTE, while in the variant, the distance between the Cα of Glu263 and Phe132 was 18.52 Å. Nevertheless, Leu271is a hydrophobic residue, and Glu263 is a negatively charged residue. The changes discussed above may influence the substrate specificity between the variant and the WT PTE. 

**Table 2:** The hydrogen bonds between residue 265 and residue 273.

Hydrogen bond Donor	Hydrogen bond acceptor
Asn165:NH	Leu262 :O
Ser267:HG	Asn265:OD1
Ala268:HN	Asn265:OD1
Ser269:HN	Asn265:O
Ala270:HN	Ala266:O
Leu271:HN	Ser267:O
Leu272:HN	Ala268:O
Gly273:HN	Ser269:O

### 2.2. Docking Study

**Figure 4:**
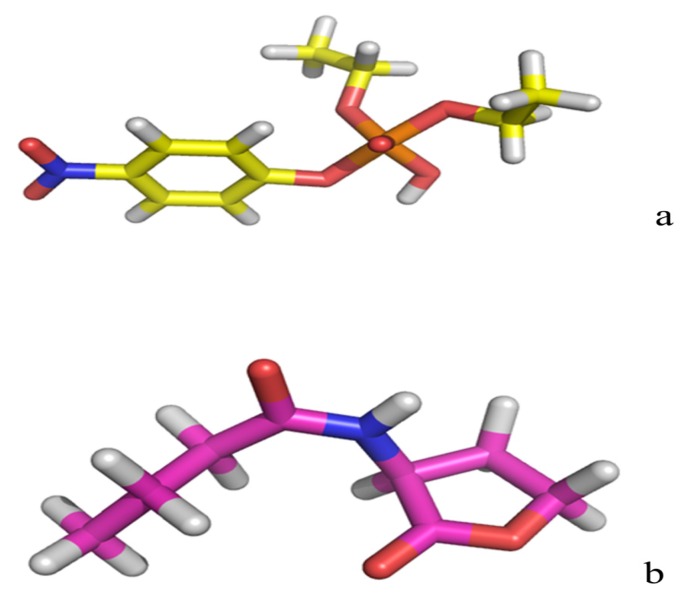
(**a**) The 3D structure of paraoxon optimized with Gaussian software at 6-31+G* set; (**b**) The 3D structure of C4-HSL optimized with Gaussian software at 6-31+G* set.

The 3D structure of paraoxon and C4-HSL are shown in Figure 4a,b. B3LYP/6-31+G* set with Gaussian software was used to calculate atomic charges for the substrates paraoxon and C4-HSL.

The docking scores are listed in Table 3. The docking score between the C4-HSL and loop 7-2/H254R variant (−6.6) was lower than that of the C4-HSL-PTE complex’s (−4.4). But the binding energy between the WT, the variant enzymes and paraoxon is very similar. Our result is consistent with the experimental data by Tawfik *et al* [24]. With the substrate paraoxon, the kinetic parameter K_cat_/k_M_(M^−1^·s^−1^) for PTE and loop 7-2-H254R were 9.08 × 10^7^ and 5.4 × 10^6^, respectively, but with C4-HSL, K_cat_/k_M_(M^−1^·s^−1^) were ≤ 9.5 and 2.2 × 10^4^, respectively [24].

**Table 3:** The docking score calculated by Autodock vina.

Protein/substrate	Paraoxon (Kcal/mol)	C4-HSL (Kcal/mol)
**PTE**	−6.1	−4.4
**loop7-2-H254R**	−5.4	−6.6

**Figure 5:**
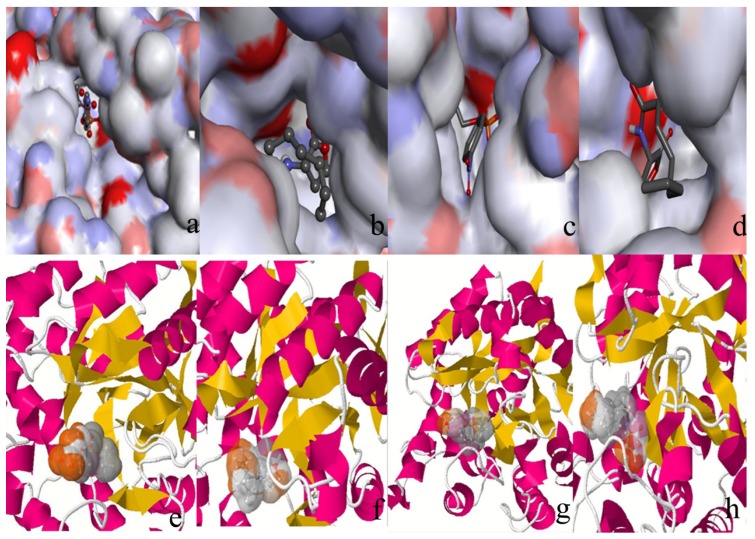
Paraoxon in the (**a**) WT PTE; (**b**) loop 7-2/H254R variant. C4-HSL in the (**c**) WT PTE; (**d**) loop 7-2/H254R variant. (**e**) Calculate and visualize molecular hydrophobic/hydrophilic properties using the concept of “Molecular Hydrophobicity Potential” (MHP). Hydrophobic and stacking interactions in paraoxon-PTE complexes can be used to re-rank the results of molecular docking. Color grey represent for a match and color brown represent for a mismatch; (**f**) Hydrophobic and stacking interactions in paraoxon-loop 7-2/H254R complexes; (**g**) Hydrophobic and stacking interactions in C4-HSL-WT PTE; (**h**) Hydrophobic and stacking interactions in C4-HSL-loop 7-2/H254R variant.

Figure 5 showed the docked complex of the enzyme and the substrates [(**a**) paraoxon-PTE; (**b**) paraoxon-loop 7-2/H254R variant; (**c**) C4-HSL-PTE; (**d**) C4-HSL-loop 7-2/H254R variant]. The substrates were all in the active pocket. Figures 5(e) and 5(h) show the calculated and visualized molecular hydrophobic/hydrophilic properties using the concept of “Molecular Hydrophobicity Potential” (MHP) with enzyme-substrate. Hydrophobic and stacking interactions in the enzyme-substrate complex can be used to re-rank the molecular docking results (in the substrate surface, the grey color represents a match and a color brown represents a mismatch). From Figure 5e and 5h, it can be concluded that all the substrates were fitted in the active pocket.

**Figure 6:**
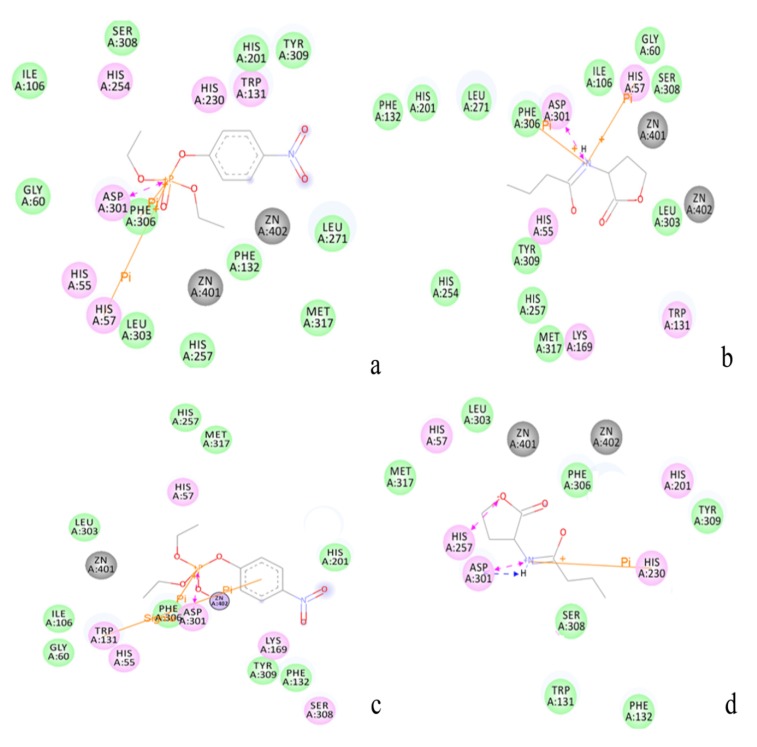
(**a**) The active residue of WT PTE around paraoxon calculated by the Discovery Studio 3.5 client. Residue interaction: pink: eletrostatic, green: van der Waals, blue: water interaction, black: metal interaction; (**b**) The active residue of loop 7-2/H254R variant around paraoxon; (**c**) The active residue of WT PTE around C4-HSL (residue number used PTE); (**d**) The active residue of loop 7-2/H254R variant around C4-HSL.

It is well known that the small binding pocket of PTE is the most isolated from the external environment compared to the large and leaving group pockets. The large binding pocket formed by His254, His257, Leu271, and Met317, is accessible to solvent molecules. The leaving group pocket is even more exposed to solvent than the large binding pocket [33]. Figure 6a,b show the active residues of the WT and the loop 7-2/H254R variant (residue number is used as WT PTE) around paraoxon. From Figure 6a,b, the phenolate substituent group was placed in the leaving group pocket (Trp131, Phe132, Phe306, and Tyr309) while the methyl and *O*-alkyl groups were positioned in the small and large pocket. Figure 6c,d indicate the active residues of the WT and the loop 7-2-H254R variant around C4-HSL. From Figure 6c,d, the aliphatic chain was placed in the leaving group pocket (Trp131, Phe132, Phe306, and Tyr309). In Figure 6, two Zn^2+^ were in all the active pockets, so the four docked complexes are reasonable and can be used for further study.

### 2.3. Protonation States of Residues His254 (Arg254) and Arg275

pKa values were calculated for all ionizable residues of PTE using the program propKa version 3.0 (Table 4) [34]. The calculations were performed for the WT and the variant form of the enzyme, namely loop 7-2-H254R. The catalytic activities of Asp233 variant were previously characterized for substrate paraoxon, and the residue was proposed to participate in the shuttling of protons from the active site [24]. By exploring possible combinations of the most probable protonation states of PTE residues, it has been found that, in addition to residues His254 and Asp301, residues Asp232, Asp235, and Arg275 take part in a hydrogen-bond network that may serve as the proton-shuffling pathway proposed by Aubert and co-workers [35]. Two residues, His254 (Arg254) and Arg275 in the active site displayed pKa values significantly shifted from their standard values. Arg275 has a little pKa value change. The pKa of His254 in the WT PTE was 6.9 units while Arg254 was 10.4 units, whereas Arg275 exhibits pKa values shifted from 9.2 units in the WT PTE to 9.4 units in the variant. It was reported that PTE displayed a maximal activity in the pH range of 9–9.5 units for the hydrolysis of paraoxon [36,37], which corresponds approximately to the estimated pKa of the Arg275. It can be inferred from the crystallographic data that both residues are involved in interactions with catalytic residues: His254 forms hydrogen bonds to Asp232 and Asp233 (In the loop 7-2/H254R variant, these hydrogens still remain) whereas Arg275 is spatially close to Asp233, near the end of a small cleft leading outward from the active site. It is reasonable to assume that the protonation states assigned to such conformation are the predominant ones at a given pH thence influence the hydrolysis of different substrates (paraoxon and C4-HSL). 

**Table 4:** Residues of the Active-Site Region with Large pKa Shifts.

Protein/substrate	His254 (R254)	Arg275
PTE	6.9	9.2
loop7-2/H254R	10.4	9.4

### 2.4. Conformational Dynamics of the PTE and the Variant upon Substrate Binding

**Figure 7:**
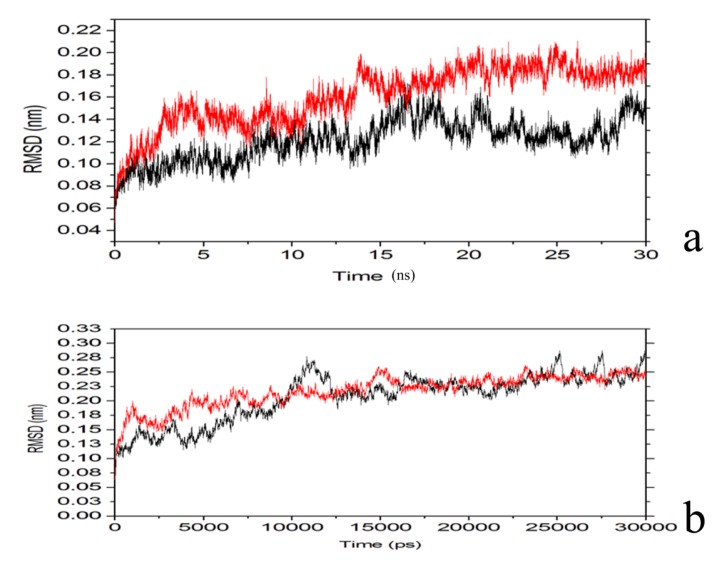
(**a**) Root-mean-square deviation (RMSD) of backbone atoms of PTE from the X-ray structure (1HYZ) for the substrate-bound state: paraoxon (black) and C4-HSL (red). (**b**) Root-mean-square deviation (RMSD) of backbone atoms of the loop 7-2/H254R variant for the substrate-bound state: paraoxon (black) and C4-HSL (red). Values are averaged for the two monomers.

Root-mean-square deviations (RMSDs) of backbone atoms of PTE from the X-ray structure (1HYZ) for the substrate-bound state: paraoxon (black) and C4-HSL (red) are shown in Figure 7. Average RMSD for the simulated systems are between 0.18 (C4-HSL (red) and 0.15 nm (paraoxon (black), reaching a first plateau around 12 ns (Figure 7a). Figure 7(b) shows the RMSD of the backbone atoms of the loop 7-2-H254R variant for the substrate-bound state: paraoxon (black) and C4-HSL (red). Average RMSD for the two simulated systems reached a first plateau around 0.23 nm, indicating a decrease of conformational flexibility upon substrate binding to the loop7-2/H254R variant. 

**Figure 8:**
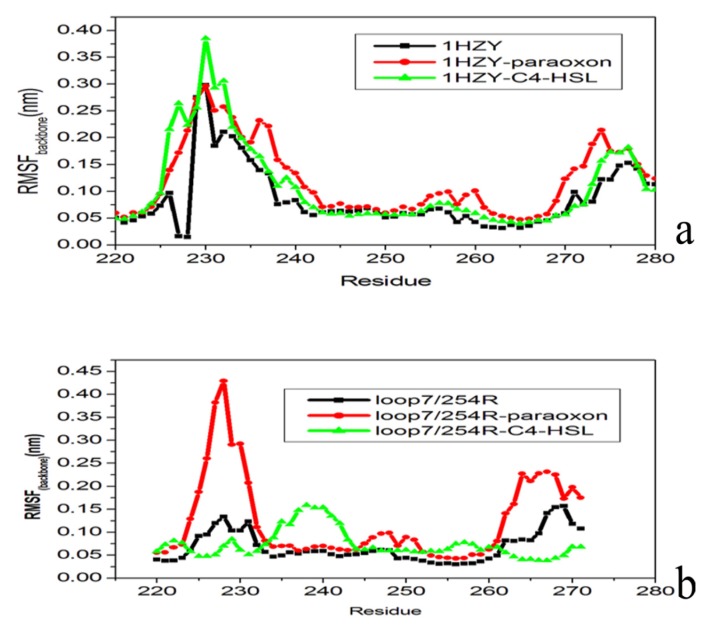
Atom-positional root-mean-square fluctuations (RMSF) of backbone atoms averaged per residue (amino acid 220 to 280) (**a**) for PTE (1HYZ) substrate free (black), substrate-bound (1HYZ- paraoxon (red) and 1HYZ-C4-HSL (green); (**b**) for loop7-2/H254 R variant substrate (black), substrate-bound (loop7-2/H254 R - paraoxon (red) and loo7-2/H254 R-C4-HSL). Values are averaged for the two OPH monomers and over 30 ns of simulations.

It has been previously shown that OPH conformational changes are mostly limited to loop residues in the entrance of the active site. Atom-positional root-mean-square fluctuations (RMSF) were calculated for backbone atoms in the WT, WT-paraoxon, and WT-C4-HSL trajectories aiming to compare the regions whose dynamics differs among these systems. Two regions comprising residues 229–239 and 263–275 exhibit rather distinct atom-positional fluctuation amplitudes for WT-C4-HSL with respect to the WT and WT- paraoxon simulations (Figure 8a). 

Residues 258-275 have been designated as loop 7 by Jackson and coworkers [38], and residues 234–239, which correspond to a preceding helix/loop motif will therefore be designated as loop 6, which contains Asp232. The large atomic fluctuations observed in the C4-HSL bound ensemble are due to the displacement of these residues toward the active site, nearly closing its cleft. These findings are further confirmed by comparison of the RMSF calculated over the entire 30 ns simulations for loop 7-2/H254R-paraoxon, loop 7-2/H254R-C4-HSL, and loop 7-2/H254R (Figure 8b). The RMSF analysis shows that the large atomic fluctuations in the loop 7-2/H254R-paraoxon simulations over the 30 ns of simulations increased significantly after the loops have closed over the active-site cleft. Such RMSF increase in these of simulations can lead to the displacement of loops 6 and 7 over the active site. It should also be noticed that the conformational rearrangement of loops 6 and 7 does not alter its secondary structure content (data not shown). These two regions are part of a loop/short-helix/loop motif located near the active site. The apparent reason for their increased flexibility is the exposure to the solvent in the MD simulations. These results indicate that, upon paraoxon binding, WT PTE undergoes a structural transition involving loops 6 and 7 located at the entrance of the active site, which allows for tighter fitting of the paraoxon substrate into the enzyme’s active site volume. While in the loop 7-2/H254R variant, the active pocket volume is almost two fold than that of the WT PTE. Thence the larger substrate, C4-HSL, can be easily released from the active site since it binds with significant conformational rearrangement at the simulated time scale. 

**Figure 9:**
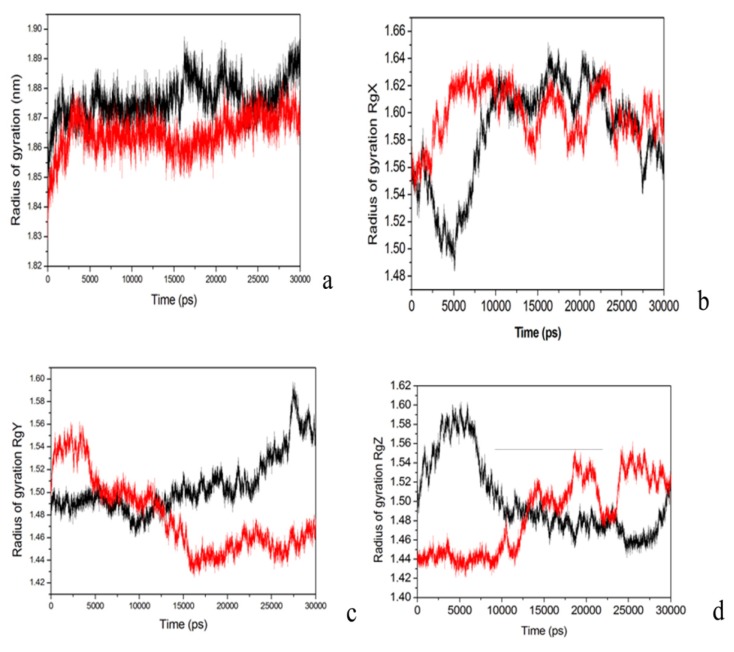
Radius of gyration (Rg) for the WT and loop 7-2/H254R variant. (**a**) WT PTE (red), loop 7-2/H254R (black); (**b**) Rg_x_ WT PTE (red), loop 7-2/H254R (black); (**c**) Rg_y_ WT PTE (red), loop 7-2/H254R (black); (**d**) Rg_z_ WT PTE (red), loop 7-2/H254R (black).

Radius of gyration (Rg) is the name of several related measures of the size of an object, a surface, or an ensemble of points. It is calculated as the root mean square distance of the objects’ parts from either its center of gravity or a given axis. Rg of the protein is represented by the volume and shape of protein. Figure 8a indicates the Rg for the WT PTE (red) and loop 7-2/H254R variant (black). The mean Rg for the WT PTE was 1.85 nm, while for the loop 7-2/H254R variant was 1.87 nm (Figure 9a). Figure 9b,c and d showed that Rg_x_, Rg_y_, and Rg_z_ direction, respectively, changes for the WT and loop 7-2/H254R variant., athough the Rg_x_, Rg_y_, and Rg_z_ were different for the variant and the WT. The mean Rg for the variant is higher than that of the WT. The season may lay in the conformational rearrangement for loop 6 and loop 7 in the variant. 

**Figure 10:**
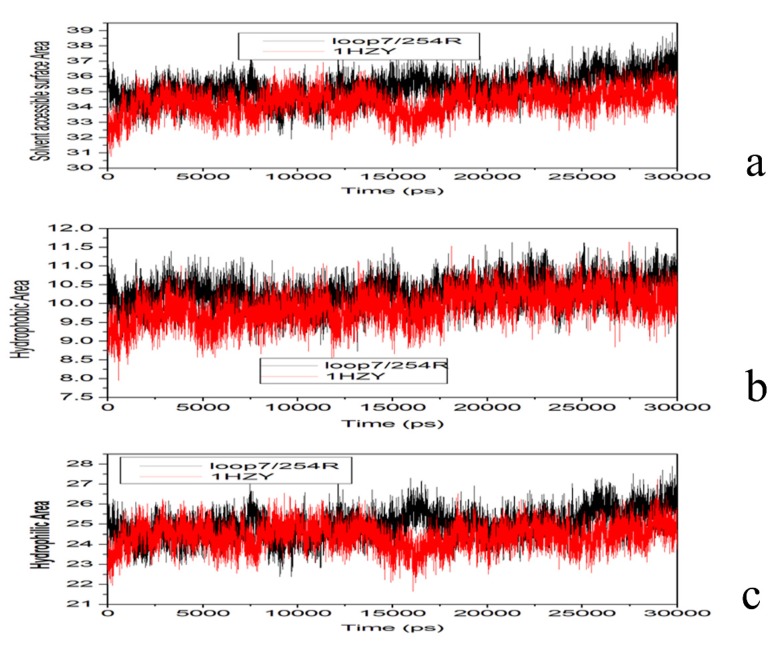
(**a**) Solvent accessible surface area. PTE (red), loop 7-2/H254R (black); (**b**) hydrophobic area. PTE (red), loop 7-2/H254R (black); (**c**) hydrophobic area. PTE (red), loop 7-2/H254R (black).

Time-dependent solvent-accessible surface area (SASA) has also been calculated for the ensemble of structures from the simulations (Figure 10a). After a period of 30 ns, the WT enzyme is smaller than that of loop 7-2/H254R variant, as well as the hydrophic area (Figure 10b) and hydrophic area (Figure 10c). Hence, C4-HSL with larger aliphatic chain can be easily released from the active site with any significant conformational rearrangement in the variant. 

**Figure 11:**
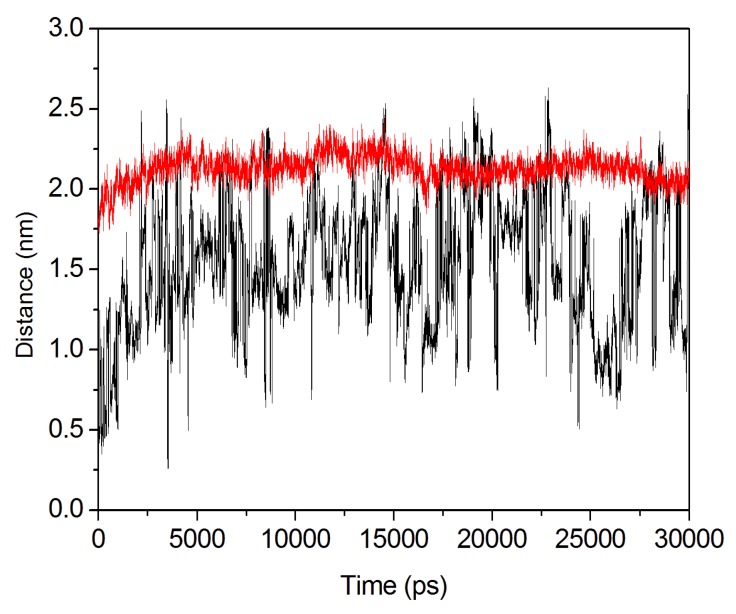
The distance between the Cα of L271 and F132 in the WT enzyme (black). The distance between the Cα of E263 and F132 in the loop 7-2/H254R (red).

It was known that Phe132 and Leu271 function as a proposed entrance gate. In the loop 7-2/H254R variant, Glu263 and Phe132 acted as the guard entrance channel. Leu271 is a hydrophobic residue, while Glu263 is a negative residue. Figure 10 shows the distance change between Phe132, Leu271 and Glu263 and Phe132 during 30 ns MD simulations. From Figure 11 it can be seen that the distance between Phe132 and Leu271 (black) varied considerably, while the distance between Glu263 and Phe132 in variant reached at 2.0 nm (red) during 30 ns simulation. The stable entrance gate distance is useful to substrates to slide in. However, it is more significant impact on C4-HSL with a large aliphatic chain than paraoxon.

## 3. Experimental

### 3.1. Computational Methodology

#### 3.1.1. Protein Preparation

High-resolution crystallographic structures of PTE from *Brevundimonas diminuta* was used as initial coordinates in the MD simulations (PDB ID 1HZY) [8]. The variant enzyme was built with Swiss model online [27]. The model was of high quality, with an above average QMEAN Z score for a protein of its size (0.74, Table 1), and further checked with Procheck [28], Verify_3D [29,30] and Errat [31]. CASTp [32] was used to identify all the cavities associated with the model and the template as well as to measure the volume they contained. Protonation states were assigned accordingly to pKa calculations with the program propKa version 3.0 [34]. The homodimers were represented identically concerning the protonation state of residues as well as the presence or absence of the substrate. 

The substrate, paraoxon and C4-HSL, were downloaded from the ChemSpider data base and then optimized at the B3LYP 6-31+G* level using the Gaussian 09 software [39,40]. Two substrates were docked in PTE and the variant type with Autodock 4.2 [41]. In the AutoDock4.2 docking process, a conformational search was performed using the Solis and Wets local search method and the Lamarckian genetic algorithm (LGA) was applied to deal with enzyme–substrate interactions. Autodock calculated enzyme-ligand interaction energies over a grid; the grid size was set to 24 × 24 × 24 Å and the grid space was the default value of 0.375 Å. The scoring function used in the energy calculations consists of electrostatic, Lennard-Jones, hydrogen bond, salvation, and torsional entropy terms. In the current version, the protein is treated as rigid while the ligand is allowed torsional flexibility. 

#### 3.1.2. Molecular Dynamics Simulation

All simulations were carried out using the GROMOS force field force parameter set 53A6 [42,43]. The active site of OPH contains two zinc ions bridged by a hydroxide anion and a carbamylated lysine. The systems were placed in a cubic box, treated for periodic boundary conditions, and solvated with explicit simple point charge (SPC) model water molecules [44]. The systems were neutralized with Na^+^ counterions where necessary. Simulations were carried out in the NPT ensemble (In the isothermal–isobaric ensemble, moles (N), pressure (P) and temperature (T) are conserved.), and a time step of 1 fs was used to integrate the equations of motion based on the Leap-Frog algorithm [45]. The temperature of the solute and solvent were separately coupled to the velocity rescale thermostat at 298.15 K with a relaxation time of 0.1 ps. The bond lengths and angles were constrained by using the P-LINCS algorithm [46], and the geometry of the water molecules was constrained using the SETTLE algorithm [47]. A twin-range cutoff of 1.0 and 1.2 nm was used for Van der Waals (VDW) interactions, and long-range electrostatic interactions were treated by the particle mesh Ewald method [48]. The systems were initially minimized through 20,000 iterations of the steepest descent algorithm. Solvent molecules were relaxed during 400 ps of simulation at 298.15 K with positional restraints applied to the heavy atoms of the protein. The full system was equilibrated for 1 ns followed by the production phase of 30 ns. Configurations of the system were recorded as trajectory files at every 1.0 ps. The software package GROMACS v.4.05 and implemented algorithms were used for all simulations and property analyses [49]. Zn^2+^ is the apparent native metal, although substantial activity is observed after substitution of the binuclear metal center by Co^2+^, Cd^2+^, Mn^2+^, or Ni^2+^[50]. Two metal ions per active site are required for full catalytic activity, and kinetic constants k_cat_ and k_cat_/k_m_ are dependent upon the identity of the specific metal cations within the active site.

## 4. Conclusions

Several long-scale molecular dynamics (MD) simulations were performed for PTE and the variant unbound and bound to two hydrolyzed substrates with very distinct catalytic efficiencies: paraoxon and C4-HSL. It has been found that: (i) there is a decrease of conformational flexibility of PTE upon substrate binding; (ii) the calculated pKa for His254 in the WT PTE and Arg254 in the variant is in the range of 6.0-10.4 units offering support to a predominantly protonated state for this residue during the enzymatic catalysis (iii) a dynamical hydrogen-bond network involving residues Asp301, His254, Asp232, Asp233, Arg275, and Asp235 may function as a pathway to shuttle protons away from the active site and (iv) molecular dynamics simulations indicate that the displacement of loops 6 and 7 over the active site in loop 7-2/H254R variant is useful to N-acyl-L-homoserine lactone (C4-HSL) with a large aliphatic chain to site in the channels easily. Our results will be helpful to further understand substrate binding in PTE.
